# Pulsed field ablation versus thermal energy ablation for atrial fibrillation: a systematic review and meta-analysis of procedural efficiency, safety, and efficacy

**DOI:** 10.1007/s10840-023-01660-3

**Published:** 2023-10-19

**Authors:** Omar Mahmoud Aldaas, Chaitanya Malladi, Frederick T. Han, Kurt S. Hoffmayer, David Krummen, Gordon Ho, Farshad Raissi, Ulrika Birgersdotter-Green, Gregory K. Feld, Jonathan C. Hsu

**Affiliations:** 1 Division of Cardiac Electrophysiology at the University of California San Diego Health System, 9452 Medical Center Drive, La Jolla, CA 92037 USA; 2 Cardiac Electrophysiology Section, Division of Cardiology, Department of Medicine, University of CA – San Diego, 9452 Medical Center Drive, 3rd Floor, Room 3E-417, La Jolla, CA 92037 USA

**Keywords:** Atrial fibrillation, Catheter ablation, Atrial fibrillation, Pulsed field ablation, Thermal ablation

## Abstract

**Background:**

Pulsed field ablation (PFA) induces cell death through electroporation using ultrarapid electrical pulses. We sought to compare the procedural efficiency characteristics, safety, and efficacy of ablation of atrial fibrillation (AF) using PFA compared with thermal energy ablation.

**Methods:**

We performed an extensive literature search and systematic review of studies that compared ablation of AF with PFA versus thermal energy sources. Risk ratio (RR) 95% confidence intervals (CI) were measured for dichotomous variables and mean difference (MD) 95% CI were measured for continuous variables, where RR < 1 and MD < 0 favor the PFA group.

**Results:**

We included 6 comparative studies for a total of 1012 patients who underwent ablation of AF: 43.6% with PFA (*n* = 441) and 56.4% (*n* = 571) with thermal energy sources. There were significantly shorter procedures times with PFA despite a protocolized 20-min dwell time (MD − 21.95, 95% CI − 33.77, − 10.14, *p* = 0.0003), but with significantly longer fluroscopy time (MD 5.71, 95% CI 1.13, 10.30, *p* = 0.01). There were no statistically significant differences in periprocedural complications (RR 1.20, 95% CI 0.59–2.44) or recurrence of atrial tachyarrhythmias (RR 0.64, 95% CI 0.31, 1.34) between the PFA and thermal ablation cohorts.

**Conclusions:**

Based on the results of this meta-analysis, PFA was associated with shorter procedural times and longer fluoroscopy times, but no difference in periprocedural complications or rates of recurrent AF when compared to ablation with thermal energy sources. However, larger randomized control trials are needed.

## Introduction

Atrial fibrillation (AF) is associated with significant morbidity and mortality [1]. Catheter ablation of AF with thermal sources such as radiofrequency or cryothermal energy has been shown to be safe and effective [2]. However, rare complications from collateral injury to adjacent structures may occur due to the indiscriminate spread of thermal energy [3]. Pulsed field ablation (PFA) uses high energy electrical impulses to induce cell death via electroporation [4, 5]. This novel energy source is more selective to cardiac myocytes and thus offers the potential advantage of delivering sufficient lesions while sparing adjacent structures, such as the esophagus, pulmonary veins, and phrenic nerve [6–9]. Until recently, most of the data on the use of PFA in the treatment of AF has been comprised of animal and single-arm studies [10]. The purpose of our current study was to perform a systematic review of the literature and meta-analysis to compare the procedural and fluoroscopy times, periprocedural complications, and recurrence of AF between PFA and thermal energy ablation in comparative studies.

## Methods

Electronic databases were searched from inception up to September 2023. No language restriction was applied. The reference list of all eligible studies was also reviewed. Search terms included (*Pulsed field ablation* OR *Electroporation*) AND (*Atrial Fibrillation OR Catheter Ablation*).

Studies were selected by two independent reviewers. The PRISMA statement for reporting systemic reviews and meta-analyses was applied to the methods for this study [11]. The studies had to fulfill the following criteria to be considered in the analysis: (1) Studies had to have compared outcomes in patients who underwent PFA with thermal ablation; (2) Studies had to have compared and reported either procedural efficiency, safety, or efficacy of the procedures; (3) Studies must have been published in a peer-reviewed scientific journal.

We aimed to compare the procedural efficiency, safety, and efficacy between PFA and thermal ablation. Two authors (O.M.A. and C.M.) independently performed literature search and extracted data from eligible studies. Outcomes were extracted from original manuscripts and supplementary data. Information was gathered using standardized protocol and reporting forms. Disagreements were resolved by consensus. Two authors (O.M.A. and C.M.) independently assessed the quality items and discrepancies were resolved by consensus or involvement of a third author (J.C.H), if necessary.

Two authors (O.M.A. and C.M.) independently assessed the risk of bias of the included trials using standard criteria defined in the Cochrane Handbook for Systematic Reviews of Interventions. Discrepancies were resolved by discussion or adjudication by a third author (J.C.H.).

Data was summarized across treatment arms using the Mantel–Haenszel risk ratio (RR), where a RR < 1.0 favored the PFA group, and inverse variance mean difference (MD), where an MD < 0 favored the PFA group. Heterogeneity of effects was evaluated using the Higgins *I*-squared (*I*^2^) statistic. Random effects models for analyses were used with high heterogeneity (defined as *I*^2^ > 25%); otherwise, fixed effects models of DerSimonian and Laird were used. Funnel plot analysis was used to address publication bias. The statistical analysis was performed by the *Review Manager (RevMan)*. *Version 5.4. Copenhagen: The Nordic Cochrane Centre, The Cochrane Collaboration, 2020.* Descriptive statistics are presented as means and standard deviations (SD) for continuous variables or number of cases (*n*) and percentages (%) for dichotomous and categorical variables.

## Results

### Study selection

**Fig. 1:**
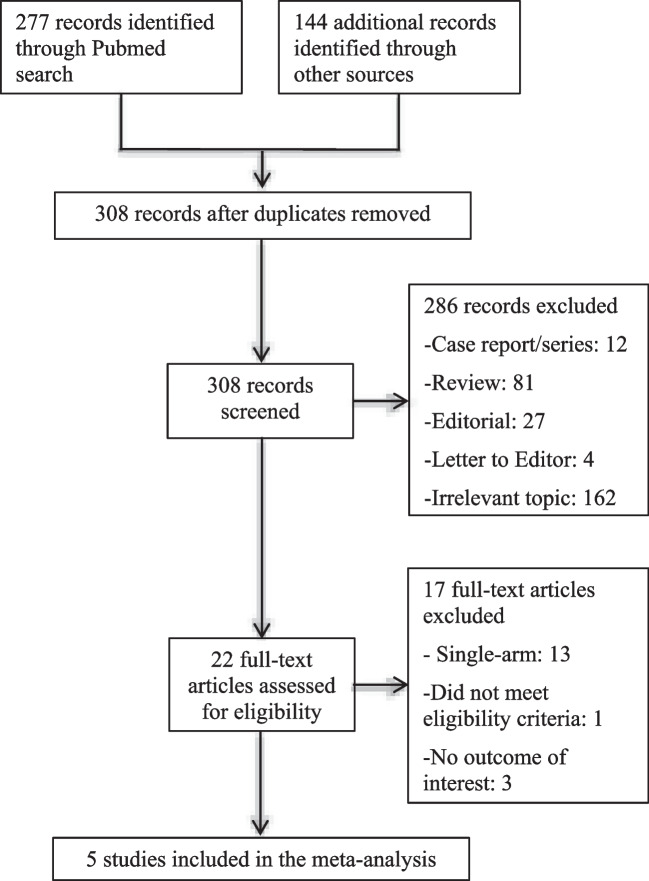
Selection of studies

The initial search resulted in 422 abstracts, of which 113 were duplications and 287 were excluded based on titles and abstracts (Fig. 1). We included six studies in our final analysis, including one randomized control trial [12], three prospective nonrandomized studies [13–16], and one retrospective study [17].

### Study characteristics

Baseline demographics of patients included in the five studies are summarized in Table 1. We included a total of 1012 patients, among which 441 (44%) underwent ablation with PFA and 571 (56%) with thermal energy sources. Patients were predominately males with paroxysmal atrial fibrillation, and many had failed anti-arrhythmic medications. None of the patients had undergone a previous ablation for AF. Study characteristics are shown in Table 2. All of the studies were prospective or single-center except for one. The Farawave™ catheter (Farapulse-Boston Scientific Inc., Menlo Park, CA, and Boston Scientific, Marlborough, MA) was used in the PFA arm of all of the studies. Among the thermal ablation patients, 56% underwent radiofrequency ablation and 44% underwent cryoablation.

**Table 1:** Patient demographics

Study	Kuroki et al. [14]		Cochet et al. [13]		Nakatani et al. [15]		Blockhause et al. [17]		Reddy et al. [12]		Maurhofer et al. [16]	
	PFA	Thermal	PFA	Thermal	PFA	Thermal	PFA	Thermal	PFA	Thermal	PFA	Thermal
Patients (*n*)	37	43	18	23	18	23	23	20	305	302	40	160
Male (*n* (%))	28 (76)	31 (72)	15 (83)	17 (74)	15 (83)	17 (74)	15 (65)	16 (80)	202 (66)	195 (65)	30 (75)	121 (76)
Age (yr)	59 ± 10	62 ± 0	58 ± 9	59 ± 9	56 ± 9	60 ± 8	57 ± 10	59 ± 9	62 ± 9	63 ± 9	62 ± 9	12 (8)
BMI	NR	NR	NR	NR	26 ± 4	26 ± 3	28 ± 4	26 ± 4	28 ± 5	29 ± 5	26 ± 4	26 ± 4
Persistent AF (*n* (%))	0 (0)	0 (0)	NR	NR	NR	NR	11 (48)	10 (50)	0 (0)	0 (0)	40 (100)	160 (100)
CHA_2_DS_2_VASc	NR	NR	NR	NR	0.5 ± 0.8	0.6 ± 0.8	1.5 ± 1.1	1.7 ± 1.4	1.7 ± 1.2	1.7 ± 1.2	NR	NR
Congestive heart failure	NR	NR	NR	NR	0 (0)	1 (4)	3 (13)	5 (25)	59 (19)	59 (20)	NR	NR
Hypertension	23 (62)	20 (47)	4 (22)	4 (17)	4 (22)	4 (17)	15 (65)	8 (40)	174 (57)	159 (53)	26 (65)	98 (61)
Diabetes mellitus	4 (11)	2 (5)	1 (6)	0 (0)	1 (6)	0 (0)	NR	NR	33 (11)	32 (11)	3 (8)	17 (11)
Stroke or TIA	2 (5)	3 (7)	2 (11)	1 (4)	1 (6)	1 (4)	0 (0)	2 (10)	12 (4)	15 (5)	2 (5)	4 (5)
CAD/vascular disease	0 (0)	6 (14)	1 (6)	2 (9)	1 (6)	2 (9)	2 (9)	5 (25)	32 (11)	51 (17)	8 (20)	24 (15)
Dyslipidemia	NR	NR	3 (17)	4 (17)	NR	NR	NR	NR	133 (44)	141 (47)	NR	NR
Ejection fraction (%)	63 ± 3	60 ± 6	62 ± 6	61 ± 8	62 ± 6	61 ± 8	56 ± 8	55 ± 8	NR*	NR*	58 ± 4	59 ± 5
Left atrial diameter (mm)	41 ± 4	38 ± 7	NR	NR	NR	NR	41 ± 3	41 ± 3	39 ± 6	40 ± 6	42 ± 6	41 ± 6
AAD (*n* (%))	NR	NR	14 (78)	17 (74)	13 (72)	18 (78)	NR	NR	185 (61)	173 (57)	NR	NR
Prior AF ablation (*n* (%))	0 (0)	0 (0)	0 (0)	0 (0)	0 (0)	0 (0)	NR	NR	0 (0)	0 (0)	0 (0)	0 (0)

Abbreviations: *AAD*, anti-arrhythmic drug; *AF*, atrial fibrillation; *BMI*, body mass index; *CAD*, coronary artery disease; *TIA*, transient ischemic attack

^*^Mean left-ventricular ejection fraction was 60% across both arms

**Table 2:** Study characteristics

Study	Kuroki et al. [14]	Cochet et al. [13]	Nakatani et al. [15]	Blockhause et al. [17]	Reddy et al. [12]	Maurhofer et al. [16]
Study design	Prospective Non-randomized Single center	Prospective Non-randomized Single center	Prospective Non-randomized Single center	Retrospective Non-randomized Single center	Prospective Randomized Multi-center	Prospective Non-randomized Single center
Study population	Patients with symptomatic paroxysmal AF resistant to AAD, LVEF > 40% and LA diameter < 5.5 cm (IMPULSE) or LA diameter < 5 cm (PEFCAT I and II)	Patients with paroxysmal AF referred for first catheter ablation procedure without contraindication to gadolinium-enhanced cardiac MRI	Patients with paroxysmal AF undergoing first catheter ablation with no contraindication to gadolinium-enhanced cardiac MRI	Patients with AF who were previously selected for pulmonary vein isolation ablation at a single center	Patients with symptomatic paroxysmal AF resistant to AAD, LVEF > 40% and LA diameter < 5.5 cm	Patients with paroxysmal AF undergoing first catheter ablation
Pulsed field ablation catheter	Farawave (Farapulse-Boston Scientific)	Farawave (Farapulse-Boston Scientific)	Farawave (Farapulse-Boston Scientific)	Farawave (Farapulse-Boston Scientific)	Farawave (Farapulse-Boston Scientific)	Farawave (Farapulse-Boston Scientific)
Pulsed field waveform	Monophasic or biphasic	Biphasic	Biphasic	NR	Biphasic	Biphasic
Type of thermal ablation	Radiofrequency	Radiofrequency in 16 (70%) and cryoablation in 7 (30%)	Radiofrequency in 16 (70%) and cryoablation in 7 (30%)	Cryoablation	Radiofrequency in 167 (55%) and cryoablation in 135 (45%)	Radiofrequency in 80 (50%) and cryoablation in 80 (50%)
Thermal ablation catheter	Contact force–sensing TactiCath catheter (St. Jude Medical) or ThermoCool NaviStar catheter (Biosense Webster)	Contact-force irrigated radiofrequency ablation catheter (Thermocool Smarttouch, Biosense Webster) or a 28-mm cryoballoon catheter (Arctic Front Advance, Medtronic)	Contact-force irrigated radiofrequency ablation catheter (Thermocool Smarttouch, Biosense Webster) or a 28-mm cryoballoon catheter (Arctic Front Advance, Medtronic)	28-mm cryoballoon catheter (2nd generation, Arctic Front Advance, Medtronic)	Saline-irrigated force-sensing radiofrequency ablation catheter or 23-mm or 28-mm cryoballoon catheter (2nd generation, Arctic Front Advance, Medtronic)	Contact-force irrigated radiofrequency ablation catheter (Thermocool Smarttouch, Biosense Webster) or a 28-mm cryoballoon catheter (Artctic Front Advance, Medtronic)
Follow-up	3 months	3 months	9 months	None	12 months	12 months
Monitoring	None	None	12-lead ECG at 1, 3, and 6 months, 24-h Holter if symptomatic	None	72-h Holter at 6 and 12 months, and trans-telephonic ECG weekly for symptoms	7-day Holter at 3, 6, and 12 months
Imaging obtained	Baseline and 3-month cardiac computed tomography scan	Baseline, within 3-h post-ablation, and 3-month cardiac magnetic resonance imaging	Baseline, within 3-h post-ablation, and 3-month cardiac magnetic resonance imaging	None	3-month cardiac computed tomography scan or magnetic resonance imaging	None

Abbreviations: *AAD*, antiarrhythmic drug; *AF*, atrial fibrillation; *ECG*, electrocardiogram; *LA*, left atrium

### Quality assessment

The risk of bias of the included observational studies is summarized in Table 3. The quality of observational studies was evaluated using the Newcastle–Ottawa Quality Assessment Scale. This scale assesses study selection, comparability, and outcomes/exposure. A good quality study will have 3–4 stars in the selection domain, 1–2 in the comparability domain, and 2–3 in the outcomes/exposure domain. A fair quality study will have 2 stars in the selection domain, 1–2 in the comparability domain, and 2–3 in the outcomes/exposure domain [18]. For the randomized control trial by Reddy et. al. [12], there was a low risk of selection bias (random sequence generation and allocation concealment), performance bias (blinding of participants), detection bias (blinding of outcome assessment), attrition bias (relatively complete outcome data), and reporting bias (all of the study’s prespecified outcomes were reported per study protocol).

**Table 3:** Newcastle–Ottawa Scale

Quality assessment criteria	Acceptable (*)	Kuroki et al. [15]	Cochet et al. [13]	Nakatani et al. [14]	Blockhause et al. [16]	Maurhofer et al. [16]
*Selection*						
Representativeness of the exposed cohort?	Truly or somewhat representative of the average patient referred for ablation	*	*	*	*	*
Selection of the non-exposed cohort?	Drawn from the same community as the exposed cohort	*	*	*	*	*
Ascertainment of exposure?	Secure record	*	*	*	*	*
Demonstration that outcome of interest was not present at start of study?	Yes	*	*	*	*	*
*Comparability*						
Study controls for antiarrhythmic drug use?	Yes	-	-	-	-	-
Study controls for at least 3 additional factors?	Age, sex, HTN, HLD, DM, CAD, CVA/TIA	-	-	-	-	*
*Outcome*						
Assessment of outcome?	Independent blind assessment or record linkage	-	-	-	-	-
Was follow-up long enough for outcomes to occur?	Yes	-	*	*	*	*
Adequacy of follow up of cohorts?	Complete follow up or subjects lost to follow up unlikely to introduce bias	*	*	*	*	*
Overall quality score (maximum = 9)		5	6	6	6	7

Abbreviations: *AF*, atrial fibrillation; *CAD*, coronary artery disease; *CVA*, cerebral vascular accident; *DM*, diabetes mellitus; *HLD*, hyperlipidemia; *HTN*, hypertension; *TIA*, transient ischemic attack

### Study endpoints

**Fig. 2:**
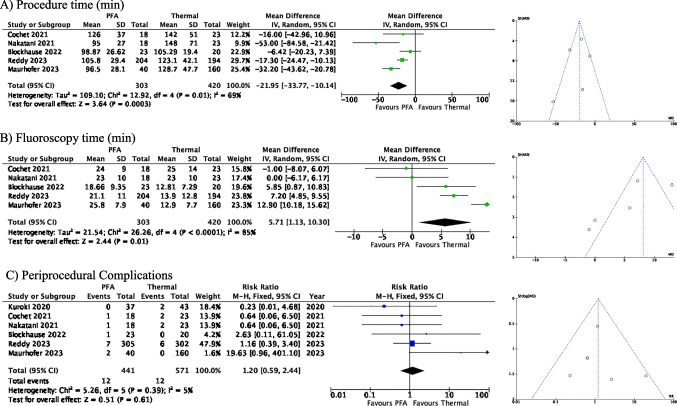
Procedural efficiency and safety outcomes in pulsed field ablation versus thermal ablation of atrial fibirllation

**Fig. 3:**
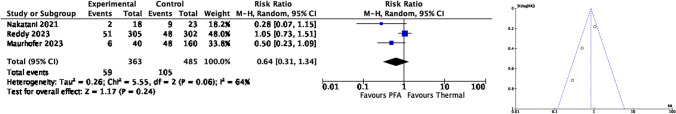
Recurrence of atrial arrhythmias at final follow-up with pulsed field ablation versus thermal ablation of atrial fibrillation

Acute procedural success was achieved in all patients except for in the study by Reddy et al. [12], where pulmonary vein isolation was achieved in 99.6% of patients in the PFA group and 99.8% in the thermal ablation group. Study end points between the PFA and thermal ablation groups are summarized in Figs. 2 and 3. There were significantly shorter procedure times with PFA despite a protocolized 20-min dwell time (MD − 21.95, 95% CI − 33.77, − 10.14, *p* = 0.0003), but with significantly longer fluoroscopy time (MD 5.71, 95% CI 1.13, 10.30, *p* = 0.01). There were no statistically significant differences in periprocedural complications (RR 1.20, 95% CI 0.59–2.44) or recurrence of atrial tachyarrhythmias (RR 0.64, 95% CI 0.31, 1.34) between the PFA and thermal ablation cohorts. Table 4 summarizes the specific periprocedural complications in both PFA and thermal ablation groups.

**Table 4:** Periprocedural complications

Study	Kuroki et al. [15]		Cochet et al. [13]		Nakatani et al. [14]		Blockhause et al. [16]		Reddy et al. [12]		Maurhofer et al. [16]	
	PFA	Thermal	PFA	Thermal	PFA	Thermal	PFA	Thermal	PFA	Thermal	PFA	Thermal
Patients (*n*)	37	43	18	23	18	23	23	20	305	302	40	160
Access site complication	NR	NR	1 (6)	2 (9)	1 (6)	2 (9)	0 (0)	0 (0)	1 (0)	2 (1)	NR	NR
Cardiac effusion or tamponade	NR	NR	0 (0)	0 (0)	NR	NR	0 (0)	0 (0)	2 (1)	0 (0)	2 (5)	0 (0)
Major bleeding	NR	NR	0 (0)	0 (0)	NR	NR	0 (0)	0 (0)	0 (0)	0 (0)	NR	NR
TIA or stroke	NR	NR	0 (0)	0 (0)	NR	NR	1 (4)	0 (0)	1 (0)	1 (0)	0 (0)	0 (0)
Coronary vasospasm	NR	NR	NR	NR	NR	NR	NR	NR	NR	NR	NR	NR
Myocardial infarction	NR	NR	0 (0)	0 (0)	NR	NR	NR	NR	0 (0)	0 (0)	NR	NR
Phrenic nerve injury	NR	NR	0 (0)	0 (0)	NR	NR	0 (0)	0 (0)	0 (0)	2 (1)	0 (0)	0 (0)
Pulmonary vein stenosis	0 (0)	2 (5)	0 (0)	0 (0)	NR	NR	NR	NR	0 (0	0 (0)	NR	NR
Atrioesophageal fistula	NR	NR	0 (0)	0 (0)	NR	NR	NR	NR	0 (0)	0 (0)	0 (0)	0 (0)
Death	NR	NR	0 (0)	0 (0)	NR	NR	0 (0)	0 (0)	1 (0)	0 (0)	0 (0)	0 (0)

Abbreviations: *TIA*, transient ischemic attack

## Discussion

This is the first systematic review and meta-analysis of comparative studies evaluating PFA versus thermal energy ablation in regard to clinical outcomes including procedural and fluoroscopy times, periprocedural complications, and recurrence of AF. The results of this meta-analysis show that ablation of AF with PFA facilitates shorter procedure times, but with longer fluoroscopy time relative to thermal ablation, with no significant differences in periprocedural complications or recurrence of AF at follow-up. The difference in procedure times is even more disparate if left atrial dwell time is instead considered, which was less than 1 h with PFA among studies that reported it [12, 17, 19]. This is despite the mandated 20-min left atrial dwell time in the PFA protocol and less operator experience with PFA catheters. Left atrial dwell time is arguably a better measure of procedure time, as sheaths are sometimes removed in the recovery area, which can lead to inaccuracies in skin-to-skin procedures times. Although there was increased fluoroscopy time in the PFA arm, this is likely explained by operator inexperience and the wide use of non-fluoroscopic, electroanatomical mapping systems with thermal ablation. Fluoroscopy time should decrease as familiarity with PFA increases and with the incorporation of mapping systems with PFA in the future [20–25].

Pulsed field ablation involves the use of micro-second, high-voltage electrical fields to cause irreversible electroporation resulting in increased cell membrane permeability and subsequent death [4, 5, 26]. The reversibility of membrane hyperpermeability is influenced by many factors, such as cell size, shape and orientation, pulse width and amplitude, number of pulses, monophasic or biphasic waveforms, pulse cycle length, and distance of the tissue from delivery electrodes [27]. PFA lesions are homogenous, preserving the extracellular matrix architecture, microvascular structures, and nerves [28, 29]. The short duration and pulses (< 100 μs) allow PFA to deliver high energy into tissues with a negligible thermal effect, potentially reducing collateral damage to surrounding tissue [5].

Despite this theoretical safety advantage with PFA, there was no significant difference in periprocedural complications seen in this meta-analysis. This could in part be explained by operator inexperience. For example, the one death reported in the PFA arm occurred due to a cardiac perforation secondary to catheter manipulation and was not related to delivery of pulsed field energy [12]. Furthermore, the higher incidence of cardiac perforation seen in some studies could be explained by the inadvertent muscle/diaphgragm twitching that can result and may be reduced as PFA waveforms are optimized [12, 30]. Additionally, special maneuvers such as esophageal deviation, temperature monitoring, and phrenic-nerve pacing have been used regularly only with thermal ablation. However, complications more common to PFA, such as coronary vasospasm, were not adequately evaluated and reported in the included studies, which is a limitation of the safety analysis and should be an outcome captured in future studies. This is even more of a concern when additional ablation lesions are delivered outside of the pulmonary veins, closer to the coronary arteries. This may have contributed to the high rates of transient hypotension and bradycardia or asystole events requiring right ventricular pacing seen with PFA in the study by Blockhaus et al. [17] However, coronary vasospasm has been shown to be subclinical in the majority of cases and is effectively treated prophylactically or post hoc with nitroglycerin. [31] Cochet et al. found no evidence of esophageal injury on post-ablation cardiac magnetic resonance imaging in patients who underwent PFA, whereas evidence of esophageal injury was noted in 10 (44%) patients who underwent thermal ablation. [13] In the studies that employed routine post-ablation cardiac imaging, the pulmonary vein ostia were narrowed to a greater extent in patients who underwent thermal ablation relative to those who had PFA ablation [12, 14]. The mechanism appears to be due to less chronic fibrosis occurring with PFA relative to thermal ablation [15]. Although there was no reported atrioesophageal fistula, pulmonary vein stenosis, or phrenic nerve injury with PFA in the included studies, the sample sizes were not powered to detect any significant differences. For example, the reported risk of atrioesophageal fistula is between 0.3 and 0.54% [32], the risk of severe pulmonary vein stenosis is between 0.32 and 3.4% [33], and the risk of phrenic nerve injury is < 1 to 6.3% after thermal ablation procedures [34, 35]. Larger scale studies with thousands of patients are needed to be powered to detect true differences in these rare complications.

Consistent with prior studies, there was no significant difference in recurrent atrial arrhythmias in patients who underwent AF ablation with PFA versus thermal ablation [36]. While it can be argued that this is expected given similar rates of acute procedural success seen between PFA and thermal ablation, the mechanisms underlying this are likely more complex. It can be hypothesized that PFA may result in more transmural lesions with less incidence of pulmonary vein reconnection, but with inadequate ablation of the adjacent ganglionated plexi due to the attenuated effect on nervous tissue, which has been implicated in the development of AF through interaction with the sympathetic and parasympathetic nervous systems [37]. This is supported by the lack of any pulmonary vein reconnections seen on repeat mapping at 3 months with PFA in the study by Nakatani et al. [15] However, in a recently published research letter by Musikantow et al. [30], late onset recurrence was largely associated with pulmonary vein reconnection rather than non-pulmonary vein triggers. Protocol-mandated invasive mapping 2–3 months after PFA performed in 110 patients revealed pulmonary vein reconnection varied by pulse waveform, with 81.9%, 16.4%, and 4.0% incidence of reconnection with monophasic, early biphasic, and optimized-biphasic waveforms, respectively. Among the 116 patients with available follow-up, 20 (17.2%) had recurrence of atrial arrhythmias within the first year. At a median post-procedural follow-up of 49 months, 85 (73%) patients remained free of atrial arrhythmias, with 79 (68%) free from atrial arrhythmias off of class I or III AAD [30]. Future studies should evaluate whether differences in catheters or waveforms could be associated with inadequate pulmonary vein isolation, or if any acute markers of reversible electroporation (acute isolation that will be at risk for chronic reconnection) can be used during the index procedure for more durable ablation with PFA. While many studies have examined the effects of different catheters, ablation duration and power settings, and lesion sets with thermal ablation, PFA is still in its nascent stage and so the optimal ablation strategy is not yet known. While the available data is promising, larger studies are needed to evaluate the safety and efficacy of PFA in the management of AF.

## Study limitations

The current systematic review and meta-analysis has several important limitations that should be acknowledged. First, the comparative studies included in the meta-analysis enrolled heterogeneous populations with variations in study design and ablation protocols, which may limit the generalizability of the results. Second, some patients may have been counted in more than one study as two of the included studies were at the same center [13, 15]. Third, there was notable heterogeneity in the use of ECGs, Holter monitors, event monitors, loop recorders, or device interrogation at various time intervals, which could have resulted in differential assessment of arrhythmia recurrence rates among studies. Fourth, all of the included studies used the same PFA catheter, which may limit the generalizability of the results. Despite these limitations, our study represents the first meta-analysis comparing AF ablation with PFA versus thermal energy sources and offers valuable data on the outcomes of PFA as a novel ablation energy source compared to the current standard of care.

## Conclusion

Based on the results of this meta-analysis, PFA compared to thermal energy ablation was associated with shorter procedural times, but with longer fluoroscopy times and no difference in periprocedural complications or rates of recurrent AF up to 1 year of follow-up. However, larger randomized controlled trials with longer follow-up comparing PFA to thermal ablation are needed.

## Abbreviations

AF: Atrial fibrillation
AAD: Antiarrhythmic drugs
CI: Confidence interval
MD: Mean difference
RR: Risk ratio
